# A Convergent Study of Genetic Variants Associated With Crohn’s Disease: Evidence From GWAS, Gene Expression, Methylation, eQTL and TWAS

**DOI:** 10.3389/fgene.2019.00318

**Published:** 2019-04-09

**Authors:** Yulin Dai, Guangsheng Pei, Zhongming Zhao, Peilin Jia

**Affiliations:** ^1^Center for Precision Health, School of Biomedical Informatics, The University of Texas Health Science Center at Houston, Houston, TX, United States; ^2^Human Genetics Center, School of Public Health, The University of Texas Health Science Center at Houston, Houston, TX, United States; ^3^Department of Biomedical Informatics, Vanderbilt University Medical Center, Nashville, TN, United States

**Keywords:** GWAS, TWAS, eQTL, integrative study, Crohn’s Disease, COP9 signalosome, *IL12RB2*, *LTBR*

## Abstract

Crohn’s Disease (CD) is one of the predominant forms of inflammatory bowel disease (IBD). A combination of genetic and non-genetic risk factors have been reported to contribute to the development of CD. Many high-throughput omics studies have been conducted to identify disease associated risk variants that might contribute to CD, such as genome-wide association studies (GWAS) and next generation sequencing studies. A pressing need remains to prioritize and characterize candidate genes that underlie the etiology of CD. In this study, we collected a comprehensive multi-dimensional data from GWAS, gene expression, and methylation studies and generated transcriptome-wide association study (TWAS) data to further interpret the GWAS association results. We applied our previously developed method called mega-analysis of Odds Ratio (MegaOR) to prioritize CD candidate genes (CDgenes). As a result, we identified consensus sets of CDgenes (62–235 genes) based on the evidence matrix. We demonstrated that these CDgenes were significantly more frequently interact with each other than randomly expected. Functional annotation of these genes highlighted critical immune-related processes such as immune response, MHC class II receptor activity, and immunological disorders. In particular, the constitutive photomorphogenesis 9 (COP9) signalosome related genes were found to be significantly enriched in CDgenes, implying a potential role of COP9 signalosome involved in the pathogenesis of CD. Finally, we found some of the CDgenes shared biological functions with known drug targets of CD, such as the regulation of inflammatory response and the leukocyte adhesion to vascular endothelial cell. In summary, we identified highly confident CDgenes from multi-dimensional evidence, providing insights for the understanding of CD etiology.

## Introduction

Crohn’s Disease (CD) is one of the major forms of inflammatory bowel disease (IBD). CD has a prevalence of 26 to 200 per 100,000 person in populations with European ancestry (Loftus, 2004). Family studies have shown that CD has 0.25 to 0.42 heritability (Gordon et al., 2015). Dysregulated immune response to environmental factors such as gut microbiome (Khor et al., 2011; Jostins et al., 2012; Ananthakrishnan, 2013) has been reported in CD. Complex diseases like CD are usually affected by a large number of genetic factors and environment factors (Rivas et al., 2011). Recent genome-wide association studies (GWAS) of CD have successfully identified more than two hundreds disease-associated loci at the genome-wide significance level (Franke et al., 2010; Liu et al., 2015). However, these findings could only explain a moderate proportion of the heritability (Verstockt et al., 2018). Recently, integrating GWAS signals with transcriptome-wide association study (TWAS) and expression quantitative trait loci (eQTL) annotation has become an effective approach to identify new susceptibility loci and has been successfully applied in several complex diseases including CD (He et al., 2013; Marigorta et al., 2017; Gusev et al., 2018). Other forms of genetic variants are also implied, such as copy number variation (CNV) and rare variants, and they are expected to have large effects (Visscher et al., 2017). For example, a genome-wide association study of CNVs identified *IRGM* (immunity-related GTPase family, M) and the HLA gene family for CD (Wellcome Trust Case Control Consortium et al., 2010). Several genes were reported to harbor rare variants associated with CD, such as *NOD2* (Nucleotide Binding Oligomerization Domain Containing 2, Alias *CARD15*) and *ADCY7* (Adenylate Cyclase 7) (Hunt et al., 2013; Luo et al., 2017). Apart from those genetic variants, epigenetic alternations were also observed in CD patients. For example, altered methylation levels in peripheral blood were reported for the genes *MIR21* (MicroRNA 21), *TXK* (TXK Tyrosine Kinase), *ITGB2* (Integrin Subunit Beta 2) and HLA loci in case-control studies (Adams et al., 2014; Ventham et al., 2016). Lastly, a number of transcriptome profiling studies have been conducted, revealing genes that were differentially expressed in CD compared to controls, such as *IFITM1* (Interferon Induced Transmembrane Protein 1), *STAT1* (Signal Transducer And Activator Of Transcription 1), *TAP1* (Transporter 1, ATP Binding Cassette Subfamily B Member), and *PSMB8* (Proteasome Subunit Beta 8) identified using endoscopic pinch biopsies (Wu et al., 2007) and *SERPINB2* (Serine (or cysteine) proteinase inhibitor, clade B (ovalbumin), member 2, PAI 2), *NCK2* (NCK Adaptor Protein 2), and *ITGB3* (Integrin Subunit Beta 3) identified using peripheral blood mononuclear cell (PBMC) (Burczynski et al., 2006). Each of these unbiased, GWAS have provided unique insights and candidate pathogenic variants and genes to understand the etiology of CD. However, challenges remain in how to effectively integrate these heterogeneous association data that range in a wide variety of biological processes.

Considerable work have been developed by integrating high-throughput multi-omics data ranging from unsupervised data integration to supervised data integration (Jiang et al., 2014; Wang et al., 2015; Huang et al., 2017; Jia et al., 2017). However, most of these tools require domain expertise, especially for the investigated diseases. Under the assumption that the number of susceptibility genes to complex disease is limited (Yang et al., 2005), we developed an unsupervised machine learning approach named mega-analysis of Odds Ratio (MegaOR) to prioritize candidate genes from multiple omics data (Jia et al., 2018). MegaOR relies on that each single omics data was conducted with control of false discoveries using the domain specific criteria (e.g., fold change for gene expression studies and stringent genome-wide significance threshold for GWAS data). We successfully demonstrated the method in schizophrenia (Jia et al., 2018). In this study, we collected five types of omics data, each representing a genome-wide association study of a molecular type with CD. We investigated the disease relevant tissues using unbiased GWAS data and conducted TWAS for CD in these tissues. By applying MegaOR, we prioritized consensus sets of candidate genes and investigated their characteristics using functional enrichment analysis and drug target crosstalk.

## Materials and Methods

### GWAS Summary Statistics

We collected the summary statistics from a GWA study for CD conducted by the International Inflammatory Bowel Disease Genetics Consortium (IIBDGC) (Liu et al., 2015). The study included 27,726 individuals (5,956 cases and 21,770 controls) of European ancestry genotyped using a combination of array platforms, including Affymetrix GeneChip Human Mapping 500K, Affymetrix Genome-Wide Human SNP Array 6.0, and Illumina HumanHap300 BeadChip. The genotype data were also imputed based on the 1000 Genomes Project reference panel (1000 Genomes Project Consortium et al., 2015). In total, the GWAS summary statistics included association results for a total of 11,002,658 SNPs either genotyped or imputed (score > 0.3).

### Gene Expression Data

We approached the gene expression data from a recent study that profiled the whole blood expression of 24 CD patients and 23 healthy controls (Ventham et al., 2016) (GEO accession ID: GSE86434). The expression data was generated using Illumina HumanHT-12 V4.0 expression BeadChip platform (GPL10558), which contained about 31,000 annotated genes with more than 47,000 probes. We used the online tool GEO2R^1^ to conduct differential gene expression analysis. We compared the expression of whole blood mRNA between CD cases and controls. Following the method used in the original paper, log2 transformation was conducted for the expression data, and then Limma (R package) was used to adjust covariates (age and gender) to obtain the differentially expressed genes (DEGs) between CD cases and controls. Genes with fold change (FC) ≥ 1.5 or ≤ 0.67 and adjusted *p-value* < 0.05 (the Benjamini and Hochberg method) were defined as DEGs (Mitra et al., 2015; Ritchie et al., 2015; Hu et al., 2018).

### Methylation Data

We obtained the methylation data from a recent study that conducted differential methylation analysis using 121 CD cases and 191 healthy controls (Ventham et al., 2016) (GEO accession ID: GSE87648). The study provided whole genome methylation using Illumina HumanMethylation450 BeadChip platform (GPL13534), which contained ∼485,000 probes. We requested the methylation results from the author of the study. This differential methylation genes was generated using whole blood leukocyte samples. In the original work (Ventham et al., 2016), the authors normalized the methylation matrix using the R package *lumi* and estimated the cell proportion by the R package *minfi*. Lastly, Limma was used to identify differentially methylated CpG probes. Probes were mapped to genes according to the annotation file of the chip (Jiang et al., 2016). For genes with multiple probes, we selected the most significant probe for the gene.

### Gene-Based Association Test Using Pascal

As our analysis builds on genes and the GWAS summary statistics provided association results for SNPs, we compiled a *p-value* for each gene using the association results of SNPs mapped to the gene. Specifically, we considered all SNPs mapped to the gene body or 50 kb upstream or downstream of the gene. We used the method Pascal to calculate the gene-based *p-values* (Lamparter et al., 2016). Pascal utilizes the sums of chi-squares and controls potential biases from gene length, SNP density, and the local LD structure. We used the European panel as the reference, as similarly, did in a recent study (Sun et al., 2018).

### Tissue-Specific Enrichment Analysis (TSEA)

To identify the tissues in which the GWAS genes were specifically expressed, we conducted a tissue specific enrichment analysis using our in-house R package, deTS (Pei et al., 2019a). deTS provides a preprocessed reference panel with 47 tissues (each with ≥ 30 samples) from the GTEx (v7) expression data (GTEx Consortium et al., 2017) and implements Fisher’s Exact Test for the enrichment analysis. We applied deTS to genes defined by the Pascal results.

### Transcriptome Wide Association Studies (MetaXcan)

Transcriptome-wide association study estimates genetically regulated expression (GReX) for each gene and conducts association studies between genes and traits by assessing the difference of GReX in trait samples and control samples. We utilized the method MetaXcan for a TWAS analysis of the CD GWAS summary statistics (Barbeira et al., 2018). The pre-calculated weight matrix was downloaded from http://predictdb.org/. We utilized three disease-relevant tissues for the analyses, where were determined based on previous knowledge and deTS results.

### Integrative Analysis of eQTL and GWAS Data (Sherlock)

Considering that many disease-associated genetic variants have regulatory roles, we applied the method Sherlock to integrate eQTLs and GWAS with the aim to identify concordant evidence between the two platforms (He et al., 2013). Sherlock uses a Bayesian statistical method to match the signature of genes from eQTLs to GWAS. As eQTL data have population and tissue specificity, we applied Sherlock for the CD GWAS data using the same tissues as for MetaXcan. A gene-based *p-value* was calculated from Sherlock for each gene in each tissue.

### Mega-Analysis of Odds Ratio (MegaOR)

**FIGURE 1:**
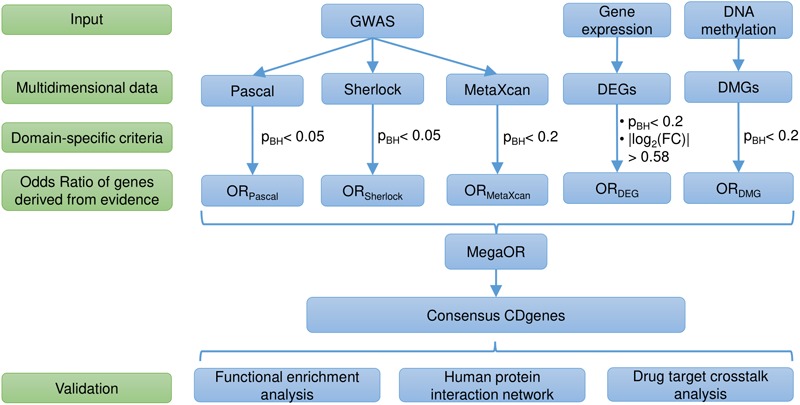
Workflow of the study. DEG, differentially expressed genes; DMG, differentially methylated genes; p_BH_, the Benjamini and Hochberg method; FC, fold change; OR, Odds Ratio; CDgenes, Crohn’s Disease genes.

We adopted our previous work MegaOR to identify a consensus set of candidate genes that collectively had the most intensive load of evidence for their association with CD (hereafter referred as CDgenes). MegaOR took a multidimensional data matrix as the input. In each dimension, genes that were determined as significantly associated with the trait based on the domain-specific threshold were labeled as 1 while other genes that failed the significance threshold were labeled as 0. For example, in the category of gene expression, significantly differentially expressed genes [FDR < 0.05 and (FC) ≥ 1.5 or ≤ 0.67] were labeled 1 and other genes 0. The same preprocessing was performed for each dimensional data following the particular domain-specific thresholds. As a result, the multidimensional data matrix included only binary values. MegaOR took this binary data matrix and defined a combined OR (cOR):cOR = μ - 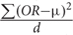, where OR represented the Odds Ratio for each dimension, *d* was the dimension of evidence, and μ was the average OR across dimensions. The part 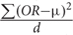 was introduced as the penalty to control deviation of any dimensional OR and served to balance the multidimensional lines of evidence. MegaOR implemented an iterative optimization procedure to find the best set of genes (denoted by *S*) with the pre-defined size *n* such that at the stable status, genes in *S* had the best cOR. A workflow was illustrated in Figure 1. Further details can be found in our previous work (Jia et al., 2018).

### Functional Enrichment Analysis

We used the R package RDAVIDWebService (version 1.16.0) for functional enrichment analysis. We focused on Gene Ontology (GO) and genetics association database (GAD) (Fresno and Fernandez, 2013). GO functional annotation tool (FAT) was used to filter out very broad terms based on a measured specificity of each term (not level-specificity). We further use the plug-in ClueGO of Cytoscape to display the relationship between genes and GO terms (Shannon et al., 2003; Bindea et al., 2009). Only GO terms with more than five CDgenes were demonstrated.

### Drug Target Gene Enrichment Analysis

We queried the Therapeutic Target Database^2^ to identify Food and Drug Administration (FDA) approved drugs that were used for CD (Li et al., 2018). Meditation target genes for CD were extracted from the database.

### Protein-Protein Interaction (PPI) Analysis

We searched the STRING database^3^ to identify protein-protein interactions (PPIs) between CD drug target genes and our CDgenes (Szklarczyk et al., 2017). We selected *Homo sapiens* as the organism and considered only the PPIs that were experimentally validated with medium confidence > 0.35.

## Results

### Multi-Dimensional Evidence for Crohn’s Disease

Using the approaches described in methods, we organized our data into five major categories: Pascal (combined GWAS information), Sherlock (integrative information of GWAS and eQTL), MetaXcan (TWAS), gene expression (with DEGs labeled as 1), and methylation (with differentially methylated genes (DMGs) labeled as 1). Particularly for Sherlock and MetaXcan, the analyses were performed for different tissues and thus, each had multiple sets of omics data. Each dimension presents a unique biological aspect to assess the potential association between a gene and CD.

**FIGURE 2:**
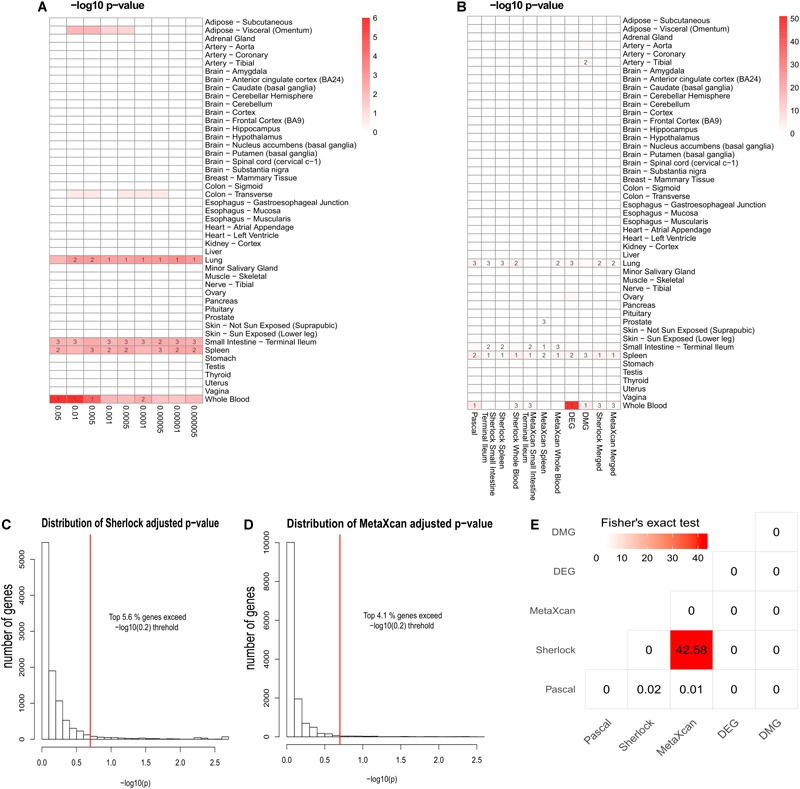
Summary of multidimensional data. **(A)** Tissue-Specific Enrichment Analysis (TSEA) of PASCAL genes. *X-axis*: groups of genes defined according to different threshold based on Pascal *p-value*. *Y-axis*: 47 GTEx tissues used as the reference panel. Top three significant tissues (adjusted *p* < 0.05 from Fisher’s Exact Test) were marked in numbers. **(B)** TSEA of genes from each gene sets. **(C)** Distribution of MetaXcan adjusted *p-value*. *X-axis*: –log10 MetaXcan adjusted *p-values* from each of the three disease-relevant tissues [whole blood, spleen and small intestine (terminal ileum)]. Red-line indicates the –log_10_ (0.2) threshold. **(D)** Distribution of Sherlock adjusted *p-values*. *X-axis*: –log10 Sherlock adjusted *p-values* from each of the three disease-relevant tissues [whole blood, spleen and small intestine (terminal ileum)]. Red-line indicates the –log_10_ (0.2) threshold. **(E)** Pair-wise comparison among the five lines of evidence. Fisher’s Exact Test was used for the significance test. The values in each cell represent the –log10 *p-value*. The figure was based on 1,668 genes that had at least one line of evidence.

As previously reported, interpretation of disease-associated genetic variants are more appropriate in tissues that are related to the diseases, as genetic regulation has a strong tissue specificity. To determine the disease-relevant tissues to CD, we conducted TSEA using the CD GWAS data (see the section “TSEA to determine CD related tissues”) and determined three tissues for the analysis of Sherlock and MetaXcan: whole blood (the most significant *p-value* was 9.75 × 10^-7^), spleen (*p* = 4 × 10^-3^), and small intestine (terminal ileum) (*p* = 5.48 × 10^-3^) (Figure 2A). As a result, we had a total of nine groups of genes: Pascal, three groups of Sherlock results, three groups of MetaXcan, DEGs, and DMGs. For each group, we applied group-specific thresholds to select positive genes (i.e., genes to be labeled as 1 in the matrix) (Table 1). Specifically, there were 773 Pascal genes (*p*_BH_ < 0.05), 289 Sherlock genes in whole blood (*p*_BH_ < 0.2), 170 Sherlock genes in spleen (*p*_BH_ < 0.2), 108 Sherlock genes in small intestine (terminal ileum) (*p*_BH_ < 0.2), 200 MetaXcan genes in whole blood (p_BH_ < 0.2), 112 MetaXcan genes in spleen (*p*_BH_ < 0.2), 69 MetaXcan genes in small intestine (terminal ileum) (*p*_BH_ < 0.2), 282 DEGs (*p*_BH_ < 0.05 and | log2(FC)| > 0.58), and 337 DMGs (*p*_BH_ < 0.2). These data collectively nominated a total of 1,668 genes, each with at least one type of association evidence. By applied TSEA to each gene sets (Figure 2B), we found that whole blood, spleen, lung, and small intestine (terminal ileum) were the most enriched tissues. Specifically, Pascal genes (*p* = 1.44 × 10^-6^), DEGs (*p* = 8.05 × 10^-52^), and DMGs (*p* = 5.82 × 10^-5^) were all most significantly enriched in whole blood. Six gene sets were most significantly enriched in spleen: the three Sherlock gene sets, MetaXcan genes calculated using small intestine (terminal ileum) and MetaXcan genes calculated using whole blood, and the merged gene set of Sherlock genes.

**Table 1:** Summary of genes from nine lines of evidence for Crohn’s Disease.

Evidence	Threshold^∗^	Number of genes passed threshold
Pascal	FDR < 0.05	773
Sherlock, whole blood	FDR < 0.2	289
Sherlock, small intestine (terminal ileum)	FDR < 0.2	108
Sherlock, spleen	FDR < 0.2	170
MetaXcan, whole blood	FDR < 0.2	200
MetaXcan, small intestine (terminal ileum)	FDR < 0.2	69
MetaXcan, spleen	FDR < 0.2	112
DEG	FDR < 0.05	282
	| log_2_FC| > 0.58	
DMG	FDR < 0.2	337

*^∗^FDR, false discovery rate; FC, fold change; DEG, differentially expressed genes; DMG, differentially methylated genes.*

Among the 1,668 genes, 1,287 (79.3%) genes had only one line of evidence and no gene was found with more than eight lines of evidence. We further merged the Sherlock genes from the three tissues and obtained a union of 398 Sherlock genes (5.6%, Figure 2C) for the following analysis of MegaOR. Similarly, a union of 305 MetaXcan genes (4.1%, Figure 2D) were obtained from three result sets in three tissues for MetaXcan. Collectively, these multidimensional data were organized as the input matrix with 1,668 genes in five dimensions, each representing one kind of disease association evidence. We referred this matrix as the evidence set (ES) genes.

As a control, we generated a second set of genes containing all the protein-coding genes that were expressed in the three CD related tissues, without requiring them to have at least one line of evidence in association with CD. Specifically, we obtained 13,763 protein-coding genes (GENCODE v19) that had an average RPKM (Reads Per Kilobase of transcript, per Million mapped reads) value >1 in whole blood, spleen, or small intestine (terminal ileum) (GTEx v7 data). These genes, referred as tissue set (TS) genes, were considered with very weak support for their potential association with CD. A total of 1,286 genes were shared between the TS genes and the 1,668 genes with evidence. After removing redundancy, we built a second matrix with a union of 14,065 genes (13,763 TS genes expressed in CD-relevant tissues and 1,668 genes with at least one line of evidence in association with CD). We applied MegaOR to both matrices and we expected that MegaOR could prioritize disease genes with or without the TS genes that had weak association evidence.

### TSEA to Determine CD Related Tissues

Crohn’s Disease causes inflammation of the gastrointestinal tract (Fakhoury et al., 2014). Digestive tissues such as colon and small intestine (terminal ileum) have long been considered to be related to CD (Wu et al., 2007). Among the multidimensional data and methods, Sherlock and MetaXcan both require pre-defined disease relevant tissues. DEGs and DMGs were obtained using blood samples. Hence, only Pascal genes from GWAS data were suitable for the determination of tissues (Pei et al., 2019b). We performed TSEA using Pascal genes defined at different threshold (*p* < 0.05, *p* < 0.01, *p* < 5 × 10^-3^, *p* < 1 × 10^-3^, *p* < 5 × 10^-4^, *p* < 1 × 10^-4^, *p* < 5 × 10^-5^, *p* < 1 × 10^-5^, and *p* < 5 × 10^-6^, Figure 2). As shown in Figure 2, Pascal genes were found to be most significantly enriched in whole blood at different thresholds (e.g., the most significant *p-value* being 9.75 × 10^-7^ when using genes with *p*_Pascal_ < 0.05), followed by small intestine (terminal ileum) (the most significant *p-value* being 3.22 × 10^-3^ when using genes with *p*_Pascal_ < 0.005). Both spleen and lung were found to be enriched with Pascal genes. However, considering that spleen acted as a filter for blood as part of the immune system while lung had no obvious link to CD, we selected whole blood, small intestine (terminal ileum), and spleen as the three most relevant tissues to CD and used these tissues for the application of Sherlock and MetaXcan.

### Pair-Wise Comparison of the Multidimensional Association Data

To explore the correlation among different dimensional data, we conducted a pair-wise comparison using genes from each group. We used Fisher’s exact test to test if any two types of evidence were associated. As shown in Figure 2E, among all possible pairs (*n* = 15), we only observed a significant correlation between Sherlock and MetaXcan genes (*p* = 2.63 × 10^-43^). This is within expectation because both data types measure the integrative signals of genetic variants and their regulatory roles in diseases. Surprisingly, Pascal genes had no correlation with either Sherlock genes (*p* = 0.95) or MetaXcan (*p* = 0.98), even though both Sherlock and MetaXcan used the same GWAS data as the input to calculate gene-based *p-values*. This lack of association implied that there was independent information that could be obtained by integrating eQTL and GReX in interpreting GWAS data, providing a fundamental support to our work of integrating these diverse evidence data. In addition, DEGs and DMGs showed no association with any of the other dimensional data.

### CDgenes Identified by MegaOR

**FIGURE 3:**
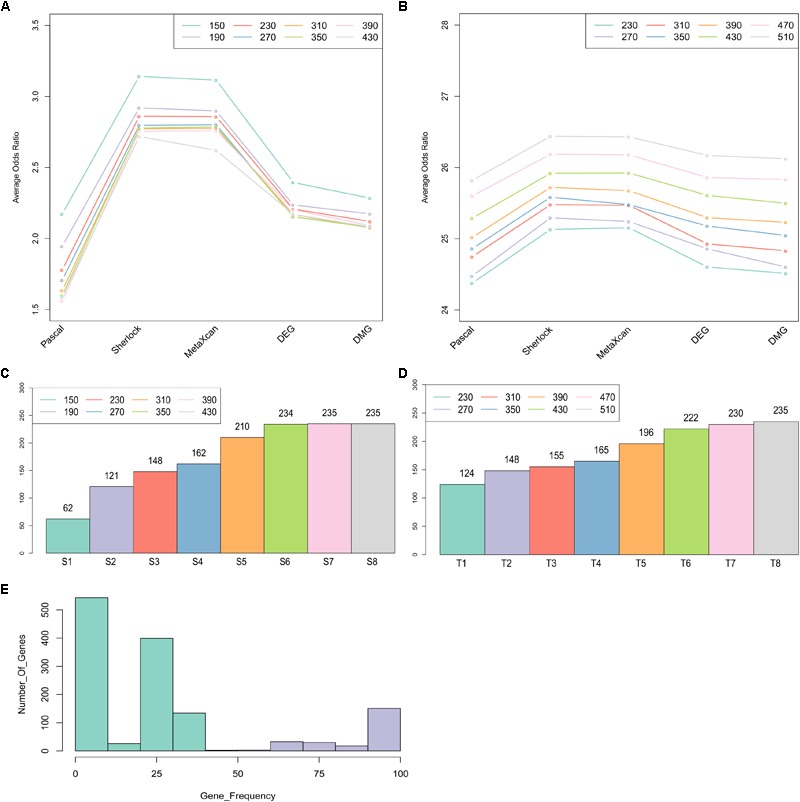
Summary of Crohn’s Disease candidate genes from MegaOR. **(A)** Odds Ratio (OR) distribution for each type of evidence in each set size for the evidence matrix with 1,668 genes. Each dot indicates the average ORs in the corresponding evidence type from 100 stable sets resulted from MegaOR (see section “Mega-Analysis of Odds Ratio”). **(B)** OR distribution for the TS-matrix. **(C)** Distribution of CDgenes at each set size. **(D)** Distribution of TS-CDgenes at each set size. **(E)** The frequency of genes covered by 100 stable sets at an example size *S* = 390 in at least on type of evidence set (ES). Genes on the left part of the plot in green were less frequently recovered (<50% occurrence). Genes on the right part of the plot were selected as the CDgenes for the corresponding set size.

To identify a set of candidate genes that have the most intensive load of evidence, we applied MegaOR to the multidimensional evidence data, respectively, the ES matrix with 1,668 genes (each with at least one type of evidence) and the TS matrix with 14,065 genes (the union of the genes expressed in disease-relevant tissues and genes from the ES matrix). We tested eight set sizes separately, i.e., *S* = 150, 190, 230, 270, 310, 350, 390, 430 for the ES matrix and *T* = 230, 270, 310, 350, 390, 430, 470, 510 for the TS matrix. For each set size, there were likely different sets of genes reaching the best cOR, even though they have the same number of genes. Thus, we applied MegaOR for each set size 100 times. The average ORs at each set sizes were displayed in Figure 3A,B. Taking the ES matrix as an example, we obtained eight sets of CDgenes. At each size, we selected genes that were retained in more than 50% times (Figure 3E). We referred the genes at each set size to S1 (set size: *S* = 150, CDgenes: 62), S2 (*S* = 190, CDgenes: 121), S3 (*S* = 230, CDgenes: 148), S4 (*S* = 270, CDgenes: 162), S5 (*S* = 310, CDgenes: 210), S6 (*S* = 350, CDgenes: 234), S7 (*S* = 390, CDgenes: 235), and S8 (*S* = 430, CDgenes: 235). CDgenes obtained using large set sizes covered nearly all the CDgenes obtained using lower set sizes. For example, the 121 genes in S2 included all the 62 genes in S1. For TS-set, T1 for set size *T* = 230 (CDgenes: 124), T2 for *T* = 270 (148), T3 for *n* = 310 (155), T4 for *n* = 350 (165), T5 for *n* = 390 (196), T6 for *n* = 430 (222), T7 for *n* = 470 (230), and T8 for *n* = 510 (235). In both sets, a converged stable status could be observed from S6 to S8 and T7 to T8, respectively (Figure 3C,D). Thus, we suggested that the 235 CDgenes in S7 and the 235 genes in T8 were close to consensus sets of CDgenes that could reach the global maximum load of evidence. Interestingly, the two sets of CDgenes (S7 and T8) shared 234 genes. Thus, we found MegaOR performed relatively stable to generate such consensus sets of candidate genes.

### CDgenes Interact With Each Other Significantly

**FIGURE 4:**
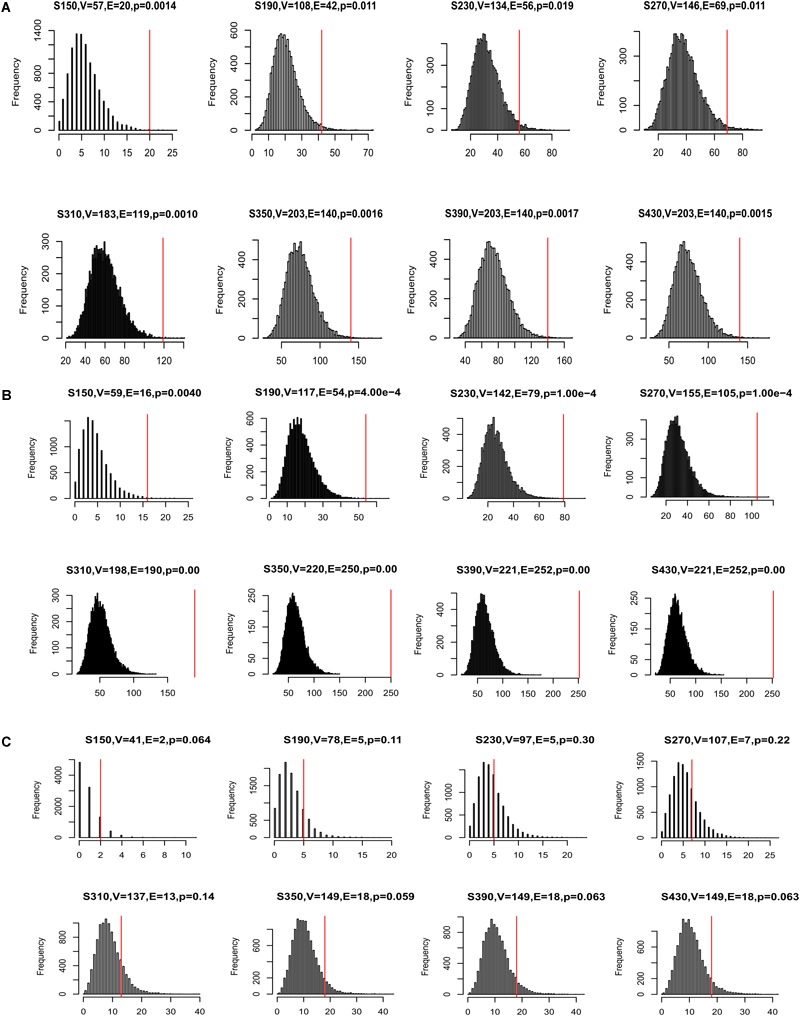
Distribution of protein-protein interactions (PPIs) among CDgenes. The analysis was conducted using the HumanNet joint reference panel **(A)**, the influence graph reference panel **(B)**, and MAGI **(C)**. In each panel, the distribution of the intersections was displayed for 10,000 randomly selected gene sets, each set with the same set sizes as the query set. *X-axis* is the number of interactions. *Y-axis* is the frequency of the interactions. In the title of each panel, V denoted the number of CDgenes that were annotated in the corresponding PPI network, E denoted the number of interactions among these CDgenes, and the *p-value* was the empirical rank *p-value*. The vertical red line indicates the number of interactions observed for the actual CDgenes.

Many disease genes were reported to interact with each other more often than with randomly selected genes, especially genes associated with the same diseases (Barabasi et al., 2011). This was likely because genes underlying the same disease are often involved in related biological pathways. To investigate whether our CDgenes tended to interact more often with each other, we curated protein-protein interaction (PPI) data from three sources. The first network was from HumanNet and has been previously used to study GWAS data (Lee et al., 2011). The second network was from a precomputed influence graph that was recently used in cancer (Ding et al., 2015). The third network was a combined dataset of HPRD and STRING (MAGI) (Hormozdiari et al., 2015). For each set of CDgenes, we recorded the number of interactions among CDgene and resampled 10,000 random gene sets, each with the same number of CDgenes. The number of random gene sets that had interactions exceeding the actual number of interactions was used to calculate an empirical *p-value*. We performed this analysis in each human PPI network, respectively. Interestingly, CDgenes showed significantly more PPIs than those from random gene sets in both HumanNet joint, influence_graph, and MAGI (Figure 4), implying that our CDgenes tended to interact with each other significantly more frequently than expected in random gene sets.

### Functional Enrichment Analysis of CDgenes

**FIGURE 5:**
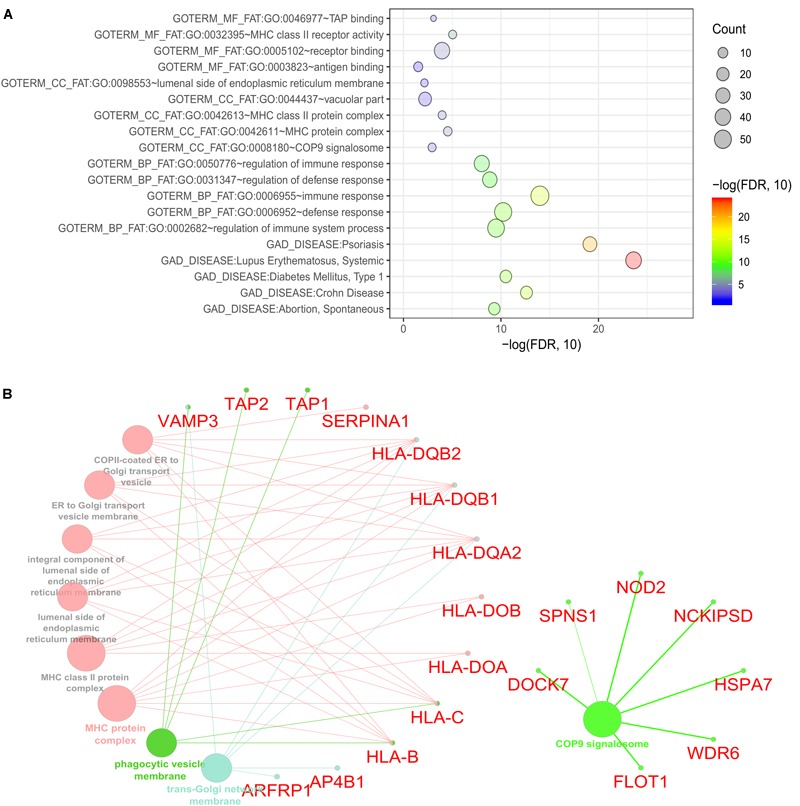
Functional enrichment for the consensus set. **(A)** Bubble plot of the functional enrichment results using the consensus CDgenes obtained at *S* = 390 (#CDgenes = 235 genes). *Y-axis* indicated the enriched gene sets with adjusted *p-value* < 0.05. *X-axis* is the –log_10_ (adjusted *p-value*). Circle sizes were proportional to the shared CDgenes with genes from the corresponding gene set. Circle color was proportional to the adjusted *p-value*. **(B)** Enrichment analysis results using the ClueGO method in Cytoscape (Bindea et al., 2009). Each dot represented a gene or a GO term. Dots in the same color were considered from the same functional group by ClueGO annotation. Gene names were highlighted in red. Each edge indicated the gene was a component gene of the linked GO term.

To identify the biological roles of the genes in the significant modules, we performed functional enrichment analysis using DAVID (See section “Materials and Methods”). We focused on GO terms and gene sets from the GAD. Our finding showed that the 235 CDgenes in S7 were enriched with MHC class II receptor activity (GO: 0032395, Molecular Function, *p* = 9.08 × 10^-6^), immune response (GO: 0006955, Biological Process, *p* = 1.02 × 10^-14^), and MHC protein complex (GO: 0042611, Cellular Component, *p* = 2.85 × 10^-5^) (Figure 5A). In GAD, immunological disorders such as Systemic Lupus Erythematosus (adjusted *p* = 2.52 × 10^-24^) and Psoriasis (adjusted *p* = 7.56 × 10^-20^) were found to be most significantly enriched (Figure 5A). Importantly, the category “Crohn’s Disease” from GAD was also significantly enriched in our CDgenes (adjusted *p* = 2.44 × 10^-13^). Evidence of 235 CDgenes was provided in Supplementary Tables S1, S2.

## Discussion

In this work, we collected five multi-dimensional data to prioritize CD-associated genes. Using tissue specific enrichment analysis and GWAS data, we determined three tissues that were most related to CD [whole blood, spleen, and small intestine (terminal ileum)]. With these tissues, we calculated integrative association signals between tissue eQTL and GWAS data and conducted tissue-specific TWAS. We constructed two evidence matrices and applied MegaOR to identify a consensus set of CD-associated genes. The candidate CDgenes in this consensus set tended to interact with each other more often than size-matched random genes, indicating these CDgenes could functionally cooperate with each other. Functional enrichment analysis showed that these CDgenes were enriched in immune related diseases and biological processes. Moreover, methods of integrative studies such as MegaOR are powerful tools to unravel the etiology of complex diseases (Wang et al., 2016; O’Brien et al., 2018). With the increasing volume of omics data, these methods could be easily extended to other complex diseases, such as cancer, psychiatric diseases, and immune diseases.

### Consensus CDgenes Overlaps With Known Disease Risk Genes

Although we did not collect the rare mutations as our evidence, two genes from our CDgenes were previously reported to harbor rare variants with CD, *ADCY7* (adjusted *p*_Pascal_ = 4.76 × 10^-10^, adjusted *p*_Sherlock_ = 2.20 × 10^-3^ in whole blood, adjusted *p*_MetaXcan_ = 9.15 × 10^-4^ in whole blood, and adjusted *p*_DMG_ = 0.045) and *NOD2* (adjusted *p*_Pascal_ = 4.76 × 10^-10^, adjusted *p*_Sherlock_ = 2.20 × 10^-3^ in whole blood, and adjusted *p*_MetaXcan_ = 0.096 in whole blood) (Hunt et al., 2013; Luo et al., 2017). Moreover, previously known DEGs and DMGs (*MIR21, TXK, IFITM1*, and *TAP1*) could also be observed in our CDgenes, suggesting these genes have robust association with CD (Adams et al., 2014; Ventham et al., 2016).

### Function Enrichment Analysis of CDgenes Highlighted COP9 Signalosome

Our consensus CDgenes provided a promising list of candidate genes for CD. The significantly enriched pathways and functional sets suggested that CDgenes were biologically related to CD. In addition, we observed quite a number of promising genes with various types of evidence, such as genes involved in antigen binding (*HLA-DOA, HLA-DOB, HLA-DQA2, HLA-DQB1, TAP1*, and *TAP2*) and genes involved in the immune response (*NOD2, IFITM1, PSMB8, TXK*, and *AIM2*). Other genes of interest included *NCKIPSD* (NCK interacting protein with SH3 domain: adjusted *p*_Pascal_ = 1.00 × 10^-3^, adjusted *p*_Sherlock_ = 0.037 in whole blood, adjusted *p*_MetaXcan_ = 0.13 in whole blood), *WDR6* (WD repeat domain 6, adjusted *p*_Pascal_ = 0.029, adjusted *p*_Sherlock_ = 5.60 × 10^-3^ in small intestine (terminal ileum), adjusted *p*_MetaXcan_ = 0.025 in whole blood), *DOCK7* (dedicator of cytokinesis 7, adjusted *p*_Pascal_ = 2.00 × 10^-3^, adjusted *p*_Sherlock_ = 2.40 × 10^-3^ in whole blood, adjusted *p*_MetaXcan_ = 2.10 × 10^-3^ in whole blood), *SPNS1* [Sphingolipid Transporter 1 (Putative), *p*_Pascal_ = 4.02 × 10^-3^, adjusted *p*_Sherlock_ = 2.19 × 10^-3^ in whole blood, *p*_MetaXcan_ = 7.43 × 10^-3^), *FLOT1* (flotillin 1, *p*_Pascal_ = 3.79 × 10^-3^, *p*_Sherlock_ = 0.13 in whole blood, *p*_DEG_ = 5.87 × 10^-3^), and *HSPA7* (encoding heat shock protein family A (Hsp70) member 7, *p*_Sherlock_ = 0.14 in whole blood, *p*_DEG_ = 1.34 × 10^-3^)]. With *NOD2*, these seven genes were all from the COP9 signalosome (CSN) (53 genes in this term from ClueGO annotation, Figure 5B and Supplementary Tables S3, S4). Interestingly, these seven genes were not the subunits of CSN complex, but they interacted with CSN complex as suggested by affinity purification and mass spectrometry experiment (Fang et al., 2008). CSN is a multi-subunit protease that regulates the activity of cullin-RING ligase (CRL) families of ubiquitin E3 complexes with isopeptidase activity. The major activities that CSN was involved included de-ubiquitination activity and phosphorylation of important signaling regulators in protein kinase activities (Wei and Deng, 2003; Wei et al., 2008). Previous studies have revealed COP9 signalosome subunit 5 (CSN5/Jab1) could regulate the development of immune system in Drosophila (Harari-Steinberg et al., 2007). In mice, deficiency of one subunit of COP9 resulted in dysfunction of paneth cell and colonic enterocyte, which could lead to impaired antimicrobial peptide and might change the composition of intestinal microbiota (Wang et al., 2014). This evidence infers the dysregulation of CSN might impact the intestinal microbiota and lead to pathogenesis of inflammatory bowel disease. In addition, disrupting CSN subunit showed impact in T-cell development and antigen response, indicating CSN might involve in the homeostasis of T cells (Menon et al., 2007; Panattoni et al., 2008). Although the debates continue on that whether microbiota, innate immunity or T cell activation leads to CD, our study shed lights on the potential etiology of CD through the dysregulation of COP9 signalosome. These seven genes were only able to be discovered when integrating multi-dimensional evidence, demonstrating the advance of MegaOR to unveil such signals, which cannot be achieved by traditional single domain approaches.

### CDgenes as the Potential Drug Target

Disease associated genes are natural candidates for drug development in both complex disease and cancer (Butcher et al., 2004; Zhao et al., 2015; Lee et al., 2016). We further compared our CDgenes with known target genes of CD meditation using the Therapeutic Targets Database (TTD) (Li et al., 2018). Overall, six FDA approved drugs were found for CD: Clofazimine, Metronidazole, Ustekinumab, MLN0002, Infliximab, and Vedolizumab. These drugs had seven target genes: *ABCB11, CYP51A1, IL12B, IL23A, ITGA4, ITGB7*, and *TNF* (Supplementary Table S5). None of them were included in our CDgenes. We queried the STRING database (See text footnote 3.) for the interactions between the seven drug target genes and the 235 CDgenes (Szklarczyk et al., 2017). We observed two CDgenes had experimental medium-confidence (>0.35) in interaction with two drug target genes: IL12RB2 (CDgene) interacting with IL12B (drug target) and LTBR (CDgene) interacting TNF (drug target) (Supplementary Figure S1). *IL12RB2* was the receptor of the drug target gene *IL12B* and was discovered from Pascal (*p* = 4.76 × 10^-10^), Sherlock (*p* = 2.19 × 10^-3^) and MetaXcan (*p* = 0.12). *LTBR* (Tumor Necrosis Factor Receptor Superfamily Member 3) was the receptor of tumor necrosis factor ligand Superfamily member 14 and was discovered from Pascal (*p* = 0.013) and Sherlock (*p* = 0.16). Moreover, two TNF Superfamily ligand genes (*TNFSF10* and *TNFSF15*) and three interleukin family genes *IL18RAP, IL27*, and *IL4* were found in our CDgenes. These findings provided some insights of our CDgenes into the identification of drug targets from multi-omics datasets.

### Limitation

There were some limitations of the current work. First, although we collected five dimensional data, there were still other omics data that were missed in our work. For example, previous studies have reported that copy number variations could be associated with CD (Wellcome Trust Case Control Consortium et al., 2010). However, the number of genes implied by CNV studies were very limited (∼10) and we could not include them into our matrix. Second, due to the limited tissue data, our DEGs and DMGs were both generated using PBMCs from CD patients and samples, instead of disease tissues from the patients. PBMCs are signs of infection and auto-immune diseases (Burczynski et al., 2006). Future studies are warranted to use samples from disease related tissues, such as intestinal biopsies (Wu et al., 2007). Lastly, due to the data heterogeneity, we used different threshold to control FDR for each individual omics data, e.g., adjusted *p* < 0.05 in selecting DEGs while adjusted *p* < 0.2 for MetaXcan, Sherlock and DMGs. This inconsistence among different omics data may lead to inaccurate estimate of the actual OR. In future studies, when more data are generated, either from different omics or multiple data sets for the same omics, an enhanced evidence matrix could be constructed to validate the current CDgenes.

## Conclusion

In summary, we conducted an integrative analysis of genetic, epigenetic, and transcriptomic data in CD. Our approach prioritized candidate genes associated with CD from multi-dimensional data and such methods could be extended to many other complex diseases with multi-dimensional omics data being available. Functional analysis of these CDgenes revealed strong immune response enrichment. We further highlighted the potential involvement of COP9 signalosome in CD and suggested interactions among our CDgenes with CD drug target genes.

## Data Availability

Publicly available datasets were analyzed in this study. The data used in R package “deTS” can be found here: https://gtexportal.org/home/. Other data could be obtained from the resource described in Materials and Methods.

## Author Contributions

PJ and ZZ conceived and designed the study. YD performed the data preparation and analysis, YD and GP performed the result demonstration. YD, PJ, and ZZ wrote the manuscript. All authors have read, edited, and approved the current version of the manuscript.

## Conflict of Interest Statement

The authors declare that the research was conducted in the absence of any commercial or financial relationships that could be construed as a potential conflict of interest.

## Footnotes

1 https://www.ncbi.nlm.nih.gov/geo/geo2r/

2 http://bidd.nus.edu.sg/group/cjttd/ (accessed 2 February 2019).

3 https://string-db.org/ (accessed 13 February 2019).

## Supplementary Material

FIGURE S1 STRING-network interaction of genes.

Table S1 Binary table for 1,688 Crohn’s Disease related genes collected from five evidence. We collected this data based on the criteria from Table 1.

Table S2 Binary table for 1,688 Crohn’s Disease related genes collected from nine evidence. We collected this data based on the criteria from Table 1.

Table S3 Binary table for seven genes shared by COP9 signalosome and Crohn’s Disease consensus genes from five evidence.

Table S4 Binary table for seven genes shared by COP9 signalosome and Crohn’s Disease consensus genes from nine evidence.

Table S5 FDA approved Crohn’s Disease drugs and their target genes obtained from Therapeutic Target Database.
