# Supplementary figures and images for: A Convergent Study of Genetic Variants Associated With Crohn’s Disease: Evidence From GWAS, Gene Expression, Methylation, eQTL and TWAS
Source: Front Genet. 2019 Apr 9;10:318. doi: 10.3389/fgene.2019.00318 (PMC6467075; doi:10.3389/fgene.2019.00318)

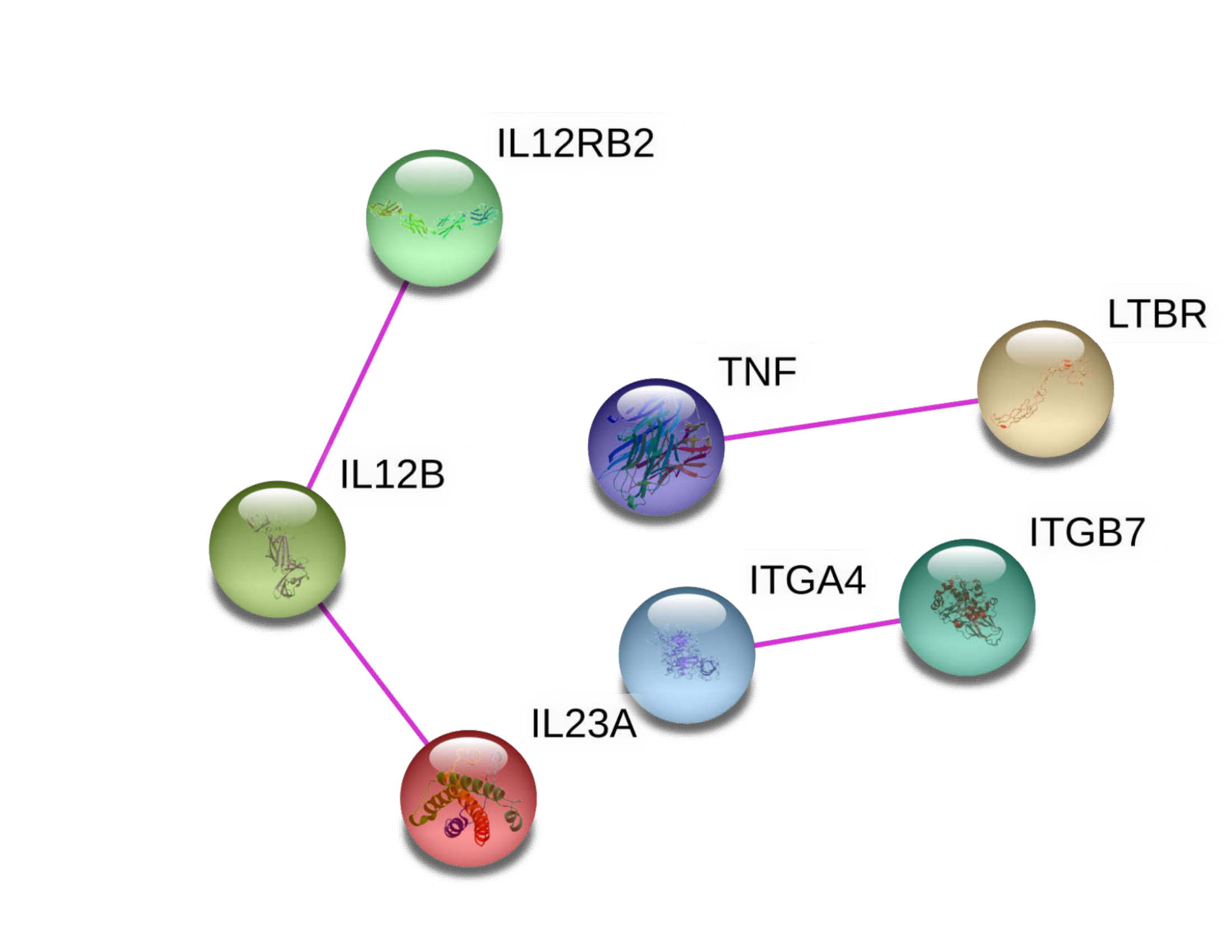

Supplement: FIGURE S1 — STRING-network interaction of genes. [file Image_1.TIFF]
